# Use of non-governmental maternity services and pregnancy outcomes among undocumented women: a cohort study from Norway

**DOI:** 10.1186/s12884-022-05112-0

**Published:** 2022-10-24

**Authors:** Frode Eick, Odd Martin Vallersnes, Heidi E. Fjeld, Ingvil Krarup Sørbye, Guro Storkås, Marthe Ekrem, Marie Børmer, Sara Andrea Løberg, Cathrine Ebbing, Nanna Voldner, Cecilie Dahl

**Affiliations:** 1 Department of Community Medicine and Global Health, Institute of Health and Society, University of Oslo, Postboks 1130 Blindern, 0318 Oslo, Norway; 2 Department of General Practice, Institute of Health and Society, University of Oslo, Oslo, Norway; 3 Department of Obstetrics, Division of Obstetrics and Gynaecology, Oslo University Hospital, Oslo, Norway; 4 Faculty of Medicine, University of Oslo, Oslo, Norway; 5 Faculty of Medicine, University of Bergen, Bergen, Norway; 6 Division of Gynaecology and Obstetrics, Akershus University Hospital, Oslo, Norway; 7 Department of Obstetrics and Gynaecology, Haukeland University Hospital, and Department of clinical medicine, University of Bergen, Bergen, Norway; 8 Faculty of Health Studies, VID specialized university, Oslo, Norway

**Keywords:** Undocumented migrants, Pregnancy, Non-governmental organizations, Antenatal care, Structural vulnerability

## Abstract

**Background:**

In 2011 Norway granted undocumented women the right to antenatal care and to give birth at a hospital but did not include them in the general practitioner and reimbursement schemes. As a response to limited access to health care, Non-Governmental Organizations (NGO) have been running health clinics for undocumented migrants in Norway’s two largest cities. To further facilitate universal health coverage, there is a need to investigate how pregnant undocumented women use NGO clinics and how this affects their maternal health. We therefore investigated the care received, occurrence of pregnancy-related complications and pregnancy outcomes in women receiving antenatal care at these clinics.

**Methods:**

In this historic cohort study we included pregnant women aged 18–49 attending urban NGO clinics from 2009 to 2020 and retrieved their medical records from referral hospitals. We compared women based on region of origin using log-binominal regression to estimate relative risk of adverse pregnancy outcomes.

**Results:**

We identified 582 pregnancies in 500 women during the study period. About half (46.5%) the women sought antenatal care after gestational week 12, and 25.7% after week 22. The women had median 1 (IQR 1–3) antenatal visit at the NGO clinics, which referred 77.7% of the women to public health care. A total of 28.4% of women were referred for induced abortion. In 205 retrieved deliveries in medical records, there was a 45.9% risk for any adverse pregnancy outcome. The risk of stillbirth was 1.0%, preterm birth 10.3%, and emergency caesarean section 19.3%.

**Conclusion:**

Pregnant undocumented women who use NGO clinics receive substandard antenatal care and have a high risk of adverse pregnancy outcomes despite low occurrence of comorbidities. To achieve universal health coverage, increased attention should be given to the structural vulnerabilities of undocumented women and to ensure that adequate antenatal care is accessible for them.

**Supplementary Information:**

The online version contains supplementary material available at 10.1186/s12884-022-05112-0.

## Background

A variety of Non-Governmental Organizations (NGOs) run health clinics for undocumented migrants to fill the gaps of limited access to primary care services in Europe [1]. While primary care is a cornerstone in securing universal health coverage and is one of the top priorities of the World Health Organization, undocumented migrants are often excluded [2–6]. To further facilitate universal health coverage for undocumented migrants, there is a need to investigate how NGO clinics contribute to primary care and interact with public services.

Migrant women’s risk profile and/or substandard antenatal care may cause increased risk of severe maternal morbidity and mortality although the effects of profile have not been substantiated [7]. Pregnancy-related complications may have long-lasting consequences for the health of both mothers and newborns; reducing maternal and newborn morbidity and mortality is essential in achieving universal health coverage for everyone living in a country, and for reaching the UN Sustainable Development Goal number 3 [8]. Literature reviews from Europe have revealed that pregnant undocumented migrants often underutilize maternal health care and, in comparison with regularized migrants, have increased risk of sexually transmitted infections, and for delivering premature babies and babies with low birth weight [9–11]. Underutilization of antenatal care may be due to restricted access, as indicated by previous studies from Norway and Denmark [12–15]. In June 2011, Norway granted pregnant undocumented women the right to antenatal care and to give birth at a hospital. However, they were excluded from the general practitioner (GP) and reimbursement schemes which are essential for access to Norwegian public health care [16, 17].

The concept of structural vulnerability has been used to describe the affects of social, economic and political structures that produce poor health and challenges in clinical care of undocumented migrants [18]. The Nordic countries are high income countries known to be inclusive welfare states to their citizens but also to be non-inclusive to undocumented migrants [19]. Undocumented migrants’ restricted social and health rights, poor working and living conditions, migratory challenges, and psychosocial hardship are well documented in Norway and in other Nordic countries [20–25]. Similarly, challenges in providing adequate clinical care to a marginalized population affected by such structural vulnerabilities have also been shown [26–30].

We know that undocumented migrants may use alternative health seeking strategies, such as approaching NGO clinics, as a response to restricted public services [13, 21, 31–33]. However, we have limited knowledge about what role NGO clinics play in primary health care, the quality of care pregnant undocumented women receive, their maternal and perinatal outcome, and if the structural vulnerabilities affect provision of universal health coverage in a Nordic setting. While studies in Nordic countries have investigated both utilization of antenatal care at such clinics and maternal outcome, these aspects have been reported separately [34–36]. Therefore, to gain a better understanding of care pathways for pregnant undocumented women, the aim of this study was to longitudinally explore the utilization of antenatal health care services at NGO clinics in the two largest cities in Norway, and to assess maternal and perinatal outcomes at three referral hospitals. We also wanted to study whether region of origin was independently associated with adverse maternal and perinatal outcomes. We hypothesized that women from the Africa and Middle East regions had worse pregnancy outcomes than women from other regions.

## Methods

### Study design and population

A historic cohort of women (aged 18–49 years) identified as pregnant in medical records from two NGO clinics were followed from their first antenatal care visit at the clinic to the end of pregnancy (abortion, or delivery at three referral hospitals between 2009 and 2020).

### Setting

The study was conducted in Oslo and Bergen, the capital and the second largest city in Norway, together comprising a population of 982,000 (18% of Norway’s total population). The two main hospitals in Oslo are Oslo University Hospital and Akershus University Hospital, and the main hospital in Bergen is Haukeland University Hospital.

Norway has a national insurance scheme, with a deductible for each consultation, for everyone who has a social security number. Undocumented migrants are excluded and have, with some exemptions such as maternity care, only right to urgent care that cannot wait [17]. GPs are mostly self-employed, while midwifes in antenatal care are mostly employed in public maternal and child health centres (MCHC). Antenatal care at MCHCs is free of charge for everyone, and secondary care is free if women cannot pay [37]. However, access may still be restricted. Any woman has the right to self-determined abortion up to 12th week of pregnancy in Norway. After gestational week 12, women need an approval from a medical tribunal.

The Norwegian Red Cross and Church City Mission have in collaboration established and are running two NGO clinics to cover the gaps in access to primary care for undocumented migrants. The NGO clinics are based on voluntarism, give free health care and medication, and, during the study period, were open for drop-in appointments 1–3 days a week. Pregnant women made up 8.8% of the patients at the NGO clinic in Oslo during the study period, whereas the proportion was not registered at the clinic in Bergen.

### Data collection

From April to June 2021, one researcher (FE) manually searched data from the medical records of all the 2135 women treated at the NGO clinics for undocumented migrants in Oslo (2009–2020) and Bergen (2014–2020). The clinic in Oslo used the electronic medical record system SOMA, and the clinic in Bergen used paper records. Information from case notes, International Classification of Primary Care, 2nd version (ICPC-2) and International Statistical Classification of Diseases and Related Health Problems, 10th revision (ICD-10) diagnoses, as well as test results from both local and supplementary investigations were collected. For haemoglobin, HIV and syphilis tests, both rapid and serology test results were in use during the period, whereas for hepatitis B only serology tests were in use. As none of the women had a social security number, they were identified in hospital records by name, maternal birth date and approximate due date. Extensive efforts to locate the records of the women at the hospitals were done from August 2021 to January 2022 by six health workers. Hospital records were used to validate and supplement information from the NGO clinics about education, marital status, parity, and planned delivery. For pregnancy outcomes, we manually searched the gynaecological and obstetric medical records in the electronic medical record system DIPS and delivery record systems PARTUS/NATUS at the three hospitals. Data from the NGO clinics and the hospitals were merged by a unique personal identity number and a pregnancy number assigned by the data collectors. Information was stored on the secure two-factor authentication TSD (Services for Sensitive Data) platform, owned and developed by the University of Oslo.

### Information collected

Country of origin was self-reported. We categorized the countries into world regions according to modified version of World Bank Regions, separating the European Economic Area from the Europe and Central Asia region [38]. Number of contacts were based on number of antenatal care registrations at the NGO clinics. We compared with World Health Organizations’ (WHO) recommendations i.e. a minimum of 8 antenatal contacts and the first antenatal contact before gestational week 12 [39]. Planned abortions were referred in the NGO records, and confirmed in the hospital records. For recommended measurements and maternal and perinatal outcome measures we compared with the definitions of the Norwegian Gynaecology Association and WHO’s ICD-10 [40, 41]. Severe postpartum bleeding was defined as recorded bleeding > 1000 ml during the first 24 hours after birth. Obstetric anal sphincter injuries were defined as 3rd and 4th degree perineal lacerations. Stillbirth was defined as a pregnancy loss at ≥22 weeks of gestation. Gestational length was calculated by both biometric second trimester ultrasonography measurements, and subsidiarily by the date of the last menstrual period. Preterm birth was defined as birth before week 37; both spontaneous and non-spontaneous. Infant low birth weight was defined as < 2500 g. Low Apgar Score was defined as < 7 at 5 min. Lost to follow up was defined as missing information on main outcome of pregnancy including spontaneous abortion, induced abortion, live birth or stillbirth.

### Patient and public involvement

Service providers from the NGO clinic in Oslo were involved in designing the study. We also discussed preliminary results with employees at the NGO clinic in Oslo and members of Humans in Limbo, a self-organized group of long-term undocumented migrants in Norway. Members of Humans in Limbo complemented the literature in understanding undocumented migrants’ structural vulnerabilities. The study was done in collaboration with service providers and will be disseminated to users, service providers, and through their work to relevant decision makers.

### Statistical analyses

Statistical analyses were performed using StataSE version 16. Descriptive characteristics of the patients and factors describing utilization of health services were summarized as number (n) and percentage (%). We used log-binominal regression to estimate relative risk (RR) with 95% confidence intervals (95% CI) of maternal and perinatal outcomes and women’s region of origin as exposure. Directed Acyclic Graphs were used to illustrate the relation between exposure (region of origin), covariates, and outcomes, and to determine which potential confounders to include in the multivariable regression [42]. We adjusted for age as the age distribution between the first assessment and the time of delivery seemed to change. The significance level was set at 0.05.

## Results

**Fig. 1:**
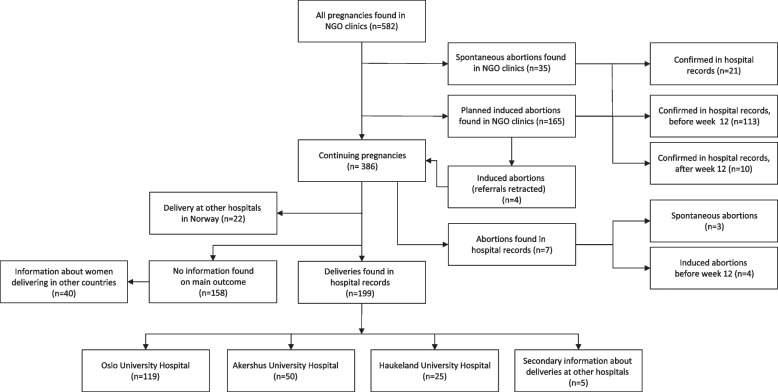
Information on pregnancies in undocumented women at two NGO clinics and three hospitals in Norway

We retrieved information from 582 pregnancies in 500 identified women who had attended one of the two NGO clinics (Fig. 1). We confirmed 74.0% (*n* = 148/200) of the planned abortions and 53.4% (*n* = 206/386) of the continuing pregnancies found at NGO clinics. Some, 10.7% (*n* = 62/582) reported an intention to deliver elsewhere, and 29.2% (*n* = 170/582) were completely lost to follow-up.

### Demographic characteristics

**Fig. 2:**
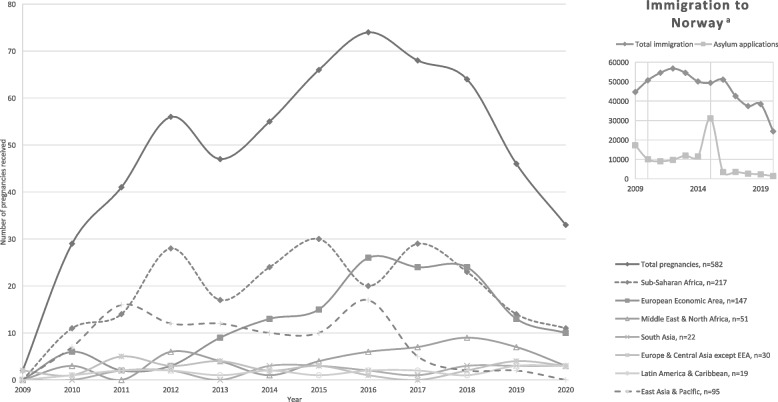
Number of pregnancies in undocumented women attending NGO clinics, by region of origin ^a^Data from Statistics Norway

The mean age of the women was 29.2 years (SD 5.7) at first antenatal visit and their median stay in Norway was 1.27 years (IQR 0.33–3.42). Most women came from Sub-Saharan Africa, 37.6% (*n* = 176/500), the European Economic Area (EEA), 24.9% (*n* = 124/500), and the East Asia & Pacific region, 16.3% (*n* = 81/500) (Fig. 2). The women originated from 73 different countries. Most women originated from Romania (*n* = 100/500), Somalia (*n* = 60/500), and Mongolia (*n* = 56/500). Of all women, 55.2% reported needing a translator in their first consultation. Compared to other regions, women from the Africa and Middle East regions had given birth to fewer children, had a longer stay in Norway, and more had applied for asylum (Table 1).

**Table 1:** Characteristics of undocumented women seeking care at NGO clinics 2009–2020 by region of origin

	Africa and Middle East regions		Other regions	
	***n*** = 222	%	***n*** = 278	%
Maternal age ^i^				
Median (years)	28.9 (IQR 25.6–33.1)		28.2 (IQR 23.3–33.1)	
Missing information	0	0	3	1.1
Education				
No school	15	6.7	2	0.7
Primary school	37	16.7	23	8.3
High school	35	15.8	40	14.4
Higher education	20	9.0	28	10.1
Missing information	115	51.8	185	66.5
Marital status				
Married/ Cohabitant	79	35.6	89	32.0
Unmarried/single/divorced	47	21.2	17	6.1
Missing information	96	43.2	172	61.9
Parity ^i^				
0	114	51.3	87	31.3
1–2	61	27.5	116	41.7
3 or more	19	8.6	30	10.8
Missing information	28	12.6	45	16.2
Pregnancies per woman during study period ^ii^				
1	186	83.8	252	90.6
2 or more	36	16.2	26	9.4
Self-reported residence status ^i^				
Asylum seeker / rejected	115	51.8	22	7.9
Not registered in Norway	36	16.2	110	39.6
Work migrant	0	0	20	7.2
Family reunification	14	6.3	15	5.4
Other	23	10.4	33	11.9
Missing information	34	15.3	78	28.0
Need of a translator				
Yes	120	54.1	156	56.1
No	58	26.1	43	15.5
Missing information	44	19.8	79	28.4
Comorbidities diagnosed				
Pre- gestational diabetes	2	0.9	2	0.7
Mental illness	9	4.1	5	1.8
Other ^iii^	6	2.7	11	3.9
Smoking at gestational start				
No	109	49.1	97	34.9
Sometimes/ daily	3	1.4	29	10.4
Missing information	110	49.5	152	54.7
Pre gestational Body Mass Index ^i, iv^				
Median	23.5 (IQR 21.7–27.0)		22.3 (IQR 20.5–25.6)	
Missing information	88	47.6	76	42.9
Years in Norway when seeking care ^i^				
Median	2.0 (IQR 0.6–3.7)		0.5 (IQR 0.2–1.5)	
Missing information	33	14.9	76	27.3
Gestational age at first antenatal visit ^i, iv^				
Median (weeks)	9 (IQR 6–19)		17 (IQR 10–26)	
Missing information	6	2.7	6	2.2

^i^At first pregnancy registered at NGO clinics

^ii^Only pregnancies registered at NGO clinics

^iii^Asthma/allergy and epilepsy

^iv^Induced abortions excluded, *n* = 362/500

### Lost to follow-up

Women lost to follow-up had a mean age of 28.8 years (SD 5.6) and the highest proportion of lost to follow up were found in age group 18–25 years (43.4%, *n* = 82/189). Women lost to follow-up originated from 52 countries, where women from the EEA had the highest proportion lost to follow up (50.3%, *n* = 74/147), in particular Romania with 51.6% (*n* = 63/122). Women lost to follow-up had a median stay in Norway of 1.16 years (IQR 0.23–3.40).

### Antenatal services

The median gestational week of the first antenatal visit at the NGO clinics was 11 (IQR 7–22), excluding women who were referred for induced abortions. About half (46.5%) presented to first antenatal visit later than gestational week 12 and 25.7% after week 22. Women from the Africa and Middle East regions presented median 8 weeks earlier for antenatal care than women from other regions (Table 1). Twelve percent of the women had become pregnant before coming to Norway. Overall, the women had a median of 1 (IQR 1–3) antenatal visit at the NGO clinics. At each antenatal visit, women met a median of 1 different midwife or doctor (IQR 1–1). Blood pressure was measured in 76.8%, and proteinuria in 57.6% of all the antenatal visits. At the NGO clinic in Oslo, use of a professional translator was documented in 21.2% (*n* = 43/203) of the first antenatal visits where a need for such a service was expressed. At the NGO clinic in Bergen, this it was not documented at all.

About half of the women (52.0%) were referred to public primary care and 17.3% to urgent or secondary public care for further antenatal care. Of the 500 women, 12.4% had more than one pregnancy during the study period (Table 1). At the NGO clinic in Bergen 77.6% (*n* = 45/58) of the women were referred to GPs working pro bono, whilst in Oslo 99.5% (*n* = 182/183) were referred for antenatal care at local MCHCs (Table 2).

**Table 2:** Characteristics of antenatal care given to undocumented women at two NGO clinics

	Bergen 2014–2020		Oslo 2009–2020	
	***n*** = 58 ^v^	%	***n*** = 359 ^**v**^	%
Number of antenatal visits at NGO clinics				
Median	1 (IQR 0–1)		2 (IQR 1–3)	
Missing information	12	20.7	12	3.3
Number of recommended measurements ^vi^				
Median	1 (IQR 0–2)		4 (IQR 2–7)	
Missing information	5	8.6	36	10.0
Documented use of professional translator at first visit ^vii^				
Yes	0	0	43	21.2
No	1	3.2	156	76.8
Missing information	30	96.8	4	2.0
Contact with public primary care before contact with NGO clinics ^viii^				
Yes	9	15.5	29	8.1
No	34	58.6	189	52.6
Missing information	15	25.9	141	39.3
Women referred to public primary care				
Maternal and child health centres	7	12.1	182	50.7
General practitioner	26	44.8	1	0.3
Missing information	1	1.7	0	0
Other follow up at public primary care	9	15.5	35	9.7
No reported follow up at public primary care	15	25.9	141	39.3
Referred to ultrasound screening week 20 ^viii^				
Yes	5	8.6	127	35.4
No	0	0	2	0.6
Missing information	53	91.4	230	64.0
Women’s need of urgent care during pregnancy				
Consultation at primary care emergency clinic	2	3.4	35	9.7
Consultation at hospital (in- or out- patient)	6	10.3	93	25.9
Women with documented post-natal check-up ^viii^				
Yes	4	6.9	50	13.9
Missing information	54	93.1	309	86.1

^v^165 (10 Bergen, 155 Oslo) induced abortions excluded (*n* = 417/582)

^vi^Performed before week 12 or at first visit; Blood pressure, haemoglobin, serum ferritin, proteinuria, hepatitis B/HIV/syphilis screening, blood type and antibody, weight, body mass index and asymptomatic bacteriological urine test

^vii^Of women reported needing translation, *n* = 234/417

^viii^Missing information may mean “No” as negative documentation is not necessarily done

The vast majority of undocumented pregnant women came to the NGO clinic in Oslo (3.84 pregnant women per month, versus 0.84 per month in Bergen). The overall trends of women seeking antenatal care peaked in 2016 and decreased thereafter but varied by region of origin (Fig. 2).

### Comorbidities and pregnancy outcome

Less than 5% of the women were diagnosed with pre-pregnancy comorbidities that may be a risk factor in pregnancy (Table 1). The overall risk of gestational diabetes was 5.8% (Table 3). Women from the Africa and Middle East regions had a higher risk of gestational diabetes, compared to women from other regions (RR 3.43, 95% CI 1.14–10.3). Overall, about half (48.9–53.5%) of the women were screened for HIV, hepatitis B and syphilis at the NGO clinics. Of those screened at the NGO clinics, 0.4% (*n* = 1/224) had a positive HIV test, 6.0% (*n* = 13/217) had a positive hepatitis B antigen test and 1.4% (*n* = 3/215) had a positive syphilis test. Table 3 shows the proportion and risk of adverse maternal and perinatal outcomes by region of origin. The risk of any adverse outcome was 45.9% for all women with a known outcome. There were no differences in risk of any adverse outcome by women’s region of origin. The overall incidence of stillbirth was 1.0%, preterm birth 10.3%, and emergency caesarean section 19.3%.

**Table 3:** Proportions and risk of adverse pregnancy outcomes among undocumented women by region of origin

	Total observations in sample	Proportion with outcome	Undocumented migrants from other regions		Undocumented migrants from Africa and Middle East		Undocumented migrants from Africa & Middle East vs. Other regions (ref.)			
	*n* = 582	%	*n* = 314	%	*n* = 268	%	RD per 1000	(95% CI)	RR ^ix^	(95% CI)
Lost to follow up ^x^	582	39.9	314	40.8	268	38.8	-20	(−99, 60)	0.96	(0.79, 1.18)
Planned induced abortion ^xi^	582	28.4	314	38.2	268	16.8	− 214	(− 284, − 144)	0.43**	(0.32, 0.58)
Confirmed induced abortion ^xii^	350	36.3	186	51.1	164	19.5	− 316	(− 410, −222)	0.38**	(0.27, 0.53)
Any adverse outcome ^xiii^	205	45.9	81	48.1	124	44.4	−38	(− 178, 102)	0.95	(0.70, 1.28)
Maternal										
Gestational diabetes	276	5.8	146	2.7	130	9.2	65	(9, 121)	3.43*	(1.14, 10.3)
Pre-eclampsia	238	2.1	119	4.2	119	0	−42*	(−78, −6)	n/a	
Severe postpartum haemorrhage	184	7.1	73	11.0	111	4.5	−65	(− 146, 17)	0.41	(0.14, 1.20)
Obstetric anal sphincter injury	184	2.2	75	2.7	109	1.8	−8	(−52, 36)	0.69	(0.10, 4.78)
Perinatal										
Stillbirth	199	1.0	79	0	120	1.7	17	(−6, 39)	n/a	
Pre-term birth, < 37w	195	10.3	77	11.7	118	9.3	−24	(−113, 65)	0.81	(0.35, 1.86)
Low birth weight, < 2500 g	199	8.0	79	8.8	120	7.5	−14	(−92, 65)	0.86	(0.34, 2.22)
Apgar score < 7 after 5 min	186	3.2	73	1.4	113	4.4	30	(−16, 77)	3.68 ^xiv^	(0.45, 30.5)
Mode of delivery										
Spontaneous vaginal	202	53.0	79	55.7	123	51.2	45	(− 185, 96)	0.90	(0.69, 1.16)
Induced vaginal	202	24.3	79	19.0	123	27.6	87	(−31, 204)	1.44	(0.84, 2.46)
Vacuum extraction	186	12.9	75	9.3	111	15.3	60	(−34, 153)	1.61	(0.70, 3.69)
Planned caesarean section	202	3.5	79	5.1	123	2.4	−26	(−81, 29)	0.88^xiv^	(0.18, 4.38)
Emergency caesarean section	202	19.3	79	20.3	123	18.7	−15	(− 128, 97)	0.92	(0.52, 1.62)

* *p*-value < 0.05

** *p*-value < 0.01

^ix^Adjusted for age. Adjusted estimates did not differ from crude, except for two outcomes, see footnote xiv

^x^On main outcome (abortion or delivery)

^xi^Data from NGO clinic records

^xii^Data from hospital records

^xiii^Severe postpartum haemorrhage > 1000ml, obstetric anal sphincter injury, stillbirth, pre term birth < 37w, lowbirth weight < 2500g, Apgar score < 7 after 5min, vacuum extraction, caesarean section

^xiv^Crude RR for Apgar score < 5 min: 3.23, Crude RR for Planned caesarean section: 0.48

### Abortions

Of the 582 pregnancies, 28.4% (*n* = 165/582) were referred for induced abortion (Table 3). Six percent (*n* = 35/582) of the pregnancies ended in a spontaneous abortion. Of the confirmed induced abortions, 8.1% were performed after gestational week 12 (Fig. 1). The number of abortions per year followed the main trends of women seeking care (Fig. 2) and had a peak in 2016 with 27 abortions. We found a difference in the proportion of induced abortion based on women’s region of origin, with a lower risk of planned, 16.8% vs. 38.2% (RR 0.43, 95% CI 0.32–0.58), and confirmed, 19.5% vs. 51.1% (RR 0.38, 95% CI 0.27–0.53), induced abortions in women from the Africa and Middle East regions than other regions (Table 3).

## Discussion

In the current study including 582 pregnancies from NGO clinics in the two largest cities in Norway, we found that about half (46.5%) of the women came in late for their first antenatal visit. We found that NGO clinics mainly serve as entry gates into public care, and by themselves do not intend to provide complete antenatal care. We found high heterogeneity in health seeking behaviour based on women’s origin, and we found a low risk (< 6%) of identified comorbidities and adverse pregnancy conditions. However, the women had a high risk of induced abortions, adverse maternal and perinatal outcomes, and sexually transmitted infections. There was also substantial loss to follow up, even with extensive efforts made to retrieve medical records from the referral hospitals.

### Women’s risk profiles

We found a low frequency of comorbidities such as pre-gestational diabetes in both women from the Africa and Middle East and other regions (0.7 and 0.9%, respectively), adverse pregnancy conditions such as gestational diabetes (9.2 and 2.7%, respectively), and few were overweight as they had median normal Body Mass Index (23.5 and 22.3, respectively). Few antenatal visits may result in a low detection of adverse pregnancy conditions, but not necessarily in a low detection of comorbidities, as women used the NGO clinics for non-pregnancy related consultations both before and during pregnancy. Few comorbidities in women may also be due to selection of those able to reach the NGO clinics or to the “healthy migrant effect”, i.e. healthier women were able to migrate or the unhealthy re-migrant effect i.e. healthier women were able to remain undocumented in Norway [43, 44]. A previous study from the two largest cities in Denmark showed a similar age distribution (average age 28.7 years) as in the current study; many were nulliparous (49.4%) and many came from Sub Saharan Africa (27.3%). However, fewer of the women came from Europe in the Danish study (13.8%) [35]. Differences in maternal outcomes for immigrant women in high income countries may be influenced not only by factors in the women’s country of origin, but also by factors in transit countries and the host country [7, 45, 46].

### Utilization of health services and care received

In the current study, a total of 46.5% of the women came late for their first antenatal visit (i.e. after the first trimester), fewer than in Denmark (52.6%) and Finland (61%) [34, 35]. Late presentation was particularly the case for women from countries outside the Africa and Middle East regions, who also had short stays in Norway. However, another study of documented migrants recently migrating (≤ 5 years) to Norway found that 16.4% came in late for their first antenatal visit [47]. Earlier studies have identified barriers to accessing health care, for example fear of deportation, financial concerns, being unfamiliar with their entitlements and not knowing where to seek help [12, 17]. In the current study, 91% reported not having been in contact with public primary care before seeking antenatal care at the NGO clinics. Among the 12.0% who became pregnant before coming to Norway, some may have attended antenatal care in their country of origin or during their migration.

The women in the study had fewer visits (1) (IQR 1–3) at the NGO clinics than the WHO recommendation of 8 visits in total. Women not referred to public primary care had similar numbers of antenatal visits as those referred. We have little information about women’s visits after referral to GPs and MCHCs, but only 52.0% of the women that came to the NGO clinics were referred to public antenatal care. Explanations for non-referral might be spontaneous abortions, women leaving Norway, late gestational attendance, or the doctor/midwife not documenting the referral. In the current study, the use of translation services, referral to ultrasound screening, screening for infectious diseases and the completion of recommended measurements were found to be low. The reason may be due to family members or volunteers translating. However, there may also be an uncertainty by the voluntary doctors and midwifes about the level of care that should be provided at the NGO clinics before and after referral to public primary care.

Our findings of the provision of substandard care should be seen in relation to the challenges of providing care to a marginalized group affected by structural vulnerabilities, on the one hand, and the NGO clinics’ limited and voluntary resources, on the other [18]. Studies from other Nordic countries show that women only attending NGO clinics, as well as those being referred to public primary care, had a low number of antenatal visits [34, 35]. A study from Sweden, found that fear, along with practical and psychosocial factors were barriers to accessing health care for undocumented migrants even when being entitled to public primary care [48]. Studies from both Denmark and Sweden have highlighted the importance of a trusting clinical relationship in the antenatal care for undocumented women [15, 49]. In our study women had to relate to a different doctor or midwife at each visit. Undocumented women in previous studies have reported feeling safe and welcome at the NGO clinics; however, they reported experiencing both positive and negative clinical encounters with public services [15, 49].

The proportion of women screened for HIV, hepatitis B and syphilis at the NGO clinics were similar to studies from Denmark (43–60%) and Finland (57–59%). The proportions of positive results in the Danish study were also similar to our study (HIV:1.5% vs. 0.4%),(hepB:6.5% vs. 6.0%),(syphilis:0% vs.1.4%) [14, 34]. An earlier study from Norway found most positive results in undocumented migrants originating from countries with high occurrence of infectious diseases [50]. Screening pregnant women for HIV, hepatitis B and syphilis is an effective method to detect infections and should be available to everyone as simple and cost-effective interventions are available to improve women’s health and prevent mother-to-child transmission.

### Maternal and perinatal outcomes

We found a 45.9% risk of any adverse outcome in pregnancy, but no difference by region of origin, despite women from the Africa and the Middle East regions attending antenatal care 8 weeks earlier than women from other regions. Adverse perinatal outcomes were frequent, with 1.0% risk of stillbirth, and 10.3% risk of preterm birth, higher than found in undocumented women in a Swedish register-based cohort study [36]. The prevalence of stillbirth in immigrants to Norway has been shown to be slightly higher than in non-immigrants (0.56% vs. 0.49%) [51]. An earlier population-based study in Norway found preterm birth rates of 6.8% in immigrants and 5.2% in non-immigrants [52]. No difference in risk of preterm birth was found by region of origin despite differences in underlying risk factors. Multiple mechanisms might initiate preterm birth [52, 53]. Studies from other populations suggest that societal factors are more important in explaining differences in preterm birth rates than genetic mechanisms [36, 54]. We found a high (19.3%) proportion of emergency caesarean section. Earlier Norwegian studies have found a 14.8% emergency caesarean rate in immigrants compared to 11.5% in non-immigrants. Disparity did not decrease with length of residence in Norway [55]. The current study revealed no difference in emergency caesarean rate by region of origin despite women from the Africa and Middle East regions staying a median of 1.5 years longer in Norway than other regions.

In the current study we found that 28.4% were referred for induced abortion and 6.0% had a spontaneous abortion. Compared to our results, pregnant undocumented women using NGO clinics in Denmark had a lower proportion of induced abortions (23 and 25.6%) and spontaneous abortion (5%) [14, 35]. An earlier study from Norway found that immigrant women had the same rate (13.6%) of induced abortions as Norwegian women in Oslo [56]. We found that 8.1% of the induced abortions were performed after week 12, a higher proportion than reported in a Danish study (3.5%) [35]. The high frequency of induced abortions indicates a need for improved access to contraceptives and potentially for family planning services.

Women from EEA and women between 18 and 25 years had the highest proportions lost to follow up. As there were fewer lost to follow up concerning abortions (26.0%) than births (46.6%), the length of time being pregnant might also have influenced whether the women were retrieved in hospital records or not. Some women (10.7%) reported that they planned to deliver in another hospital within Norway or in another country. Others might have been deported or left Norway by their own free will.

### Underlying reasons for underutilization and adverse outcomes

Our findings suggest that pregnant undocumented women underutilize and receive substandard antenatal care in Norway, despite both having the right to public antenatal care and the possibility to use NGO clinics. However, we do not have the total overview of women’s use of different primary care structures. The trend in use of NGO clinics does not seem to be directly influenced by the policy changes in Norway that gave undocumented women formal rights to antenatal care in June 2011. The use of NGO clinics increased steadily from their start in 2009 to the year 2016, coinciding with the peak of undocumented migrants in Europe in 2016 [57]. Pregnant undocumented migrants have a higher risk of adverse outcomes compared to what is found among immigrants and Norwegian born women, when comparing to previous studies [51, 52, 54–56]. Underutilization of antenatal care could explain some of the risk of adverse maternal and perinatal outcomes, but not necessarily all. We found differences in maternal conditions, parity, time stayed in Norway when seeking care, and gestational age at first antenatal visit by regions of origin that were not associated with the outcomes.

Women’s previous experiences from, and familiarity with, other health care system, socio-cultural values and practices, as well as health literacy more broadly influence the use of antenatal care [45, 58]. However, structural vulnerabilities, such as restricted access, low or no income, degrees of dependency on others, and psychosocial hardship, might have a stronger influence on their ability to access adequate antenatal care. Some of these vulnerabilities produced by individual, structural, and institutional barriers have been seen in immigrant women in Norway as well, but based on studies from elsewhere there are reasons to believe that these vulnerabilities are more deep-rooted for undocumented women [7, 10, 11, 59]. A systematic literature review found that immigrant mothers in the Nordic countries with a comprehensive integration policy had better maternal outcomes than immigrant mothers in countries with a weak integration policy [60]. However, pregnant undocumented migrants are not included in the integration policies and initiatives in Nordic countries which are ranked low when it comes to policy towards undocumented migrants [61]. The Nordic welfare states have also been described as being “soft on the inside” (for citizens), but “hard on the outside” (for undocumented migrants) [19].

It has been argued for operationalizing structural vulnerability in clinical practice and that clinicians, health systems and policy makers should counteract presumptions of deservingness in order to improve the situation for undocumented migrants [62, 63]. Initiatives elsewhere in including pregnant undocumented women in health insurance schemes, and increasing access have increased health coverage and prenatal visits, and reduced infant mortality [6, 64]. The COVID 19 pandemic has shown us the need to address structural conditions to achieve health for all [65]. Empowering marginalized groups through community mobilisation is essential for equitable and resilient health systems. This could open up a collective approach targeting the structural vulnerabilities pregnant undocumented women are facing in Nordic countries, to improve their rights and the accessibility to adequate antenatal care.

### Strengths and limitations

The strength of this study is the high number of included pregnancies (relative to previous studies in this field), and the long study period of 11 years. Cohort studies done in a pregnant undocumented migrant population are rare. With extensive efforts, we managed to retrieve hospital records from 60.1% of the women. Some women had up to 20 different hospital records with different ad hoc numbers, and it was challenging to identify and locate the information. Due to the irregular situation of the population, we chose to analyse loss to follow-up as an outcome, both to be transparent and to highlight difficulties of doing research on this population. Because of loss to follow up and missing information we have to interpret the results on maternal outcomes with caution. However, the current study describes a population that has rarely been explored. Women seeking care at the NGO clinics may be a selective group, as pregnant undocumented women may receive antenatal care elsewhere. Some of the women had additional pregnancies during the study period that were not included, as they did not seek help for these pregnancies at the NGO clinics. The quality of the information gathered must be viewed in context of what is recorded with the purpose to provide care. There may also be a problem of misclassification as part of the information is recorded based on self-report from the patients and collected by six different health care workers.

## Conclusions

Pregnant undocumented women who use NGO clinics have low occurrence of comorbidities receive substandard antenatal care. The NGO clinics served as entry gates into public health care for the vast majority of the women, yet we found that pregnant undocumented women had a high risk of adverse maternal and perinatal outcomes. Despite their right to public antenatal care and access to the NGO clinics, this study suggests that increased attention should be given to ensure the accessibility of adequate antenatal care for undocumented women. There was also a high rate of induced abortions suggesting the need for better access to contraceptives. To fulfil their commitment to universal health coverage, we argue that the Nordic countries should increase their efforts to reach undocumented women, ensure a trusting clinical relationship in antenatal care and take structural vulnerabilities into account when designing health services.

## Supplementary Information

**Additional file 1.** Directed Acyclic Graphs.

## Data Availability

The datasets generated and/or analysed during the current study are not publicly available due to the sensitivity of the data but are available from the corresponding author on reasonable request.
