# Supplementary figures and images for: Use of non-governmental maternity services and pregnancy outcomes among undocumented women: a cohort study from Norway
Source: BMC Pregnancy Childbirth. 2022 Oct 24;22:789. doi: 10.1186/s12884-022-05112-0 (PMC9589618; doi:10.1186/s12884-022-05112-0)

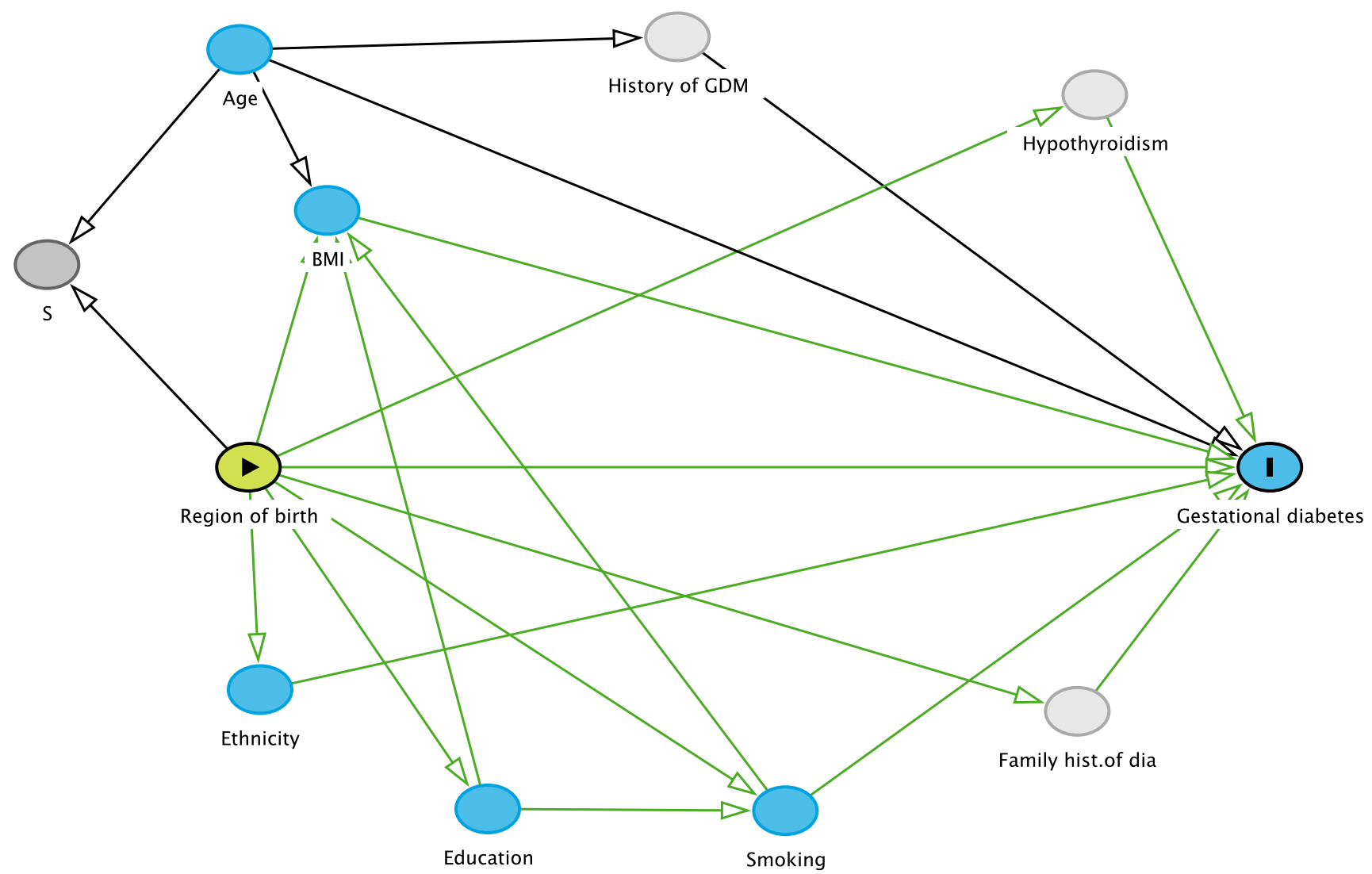

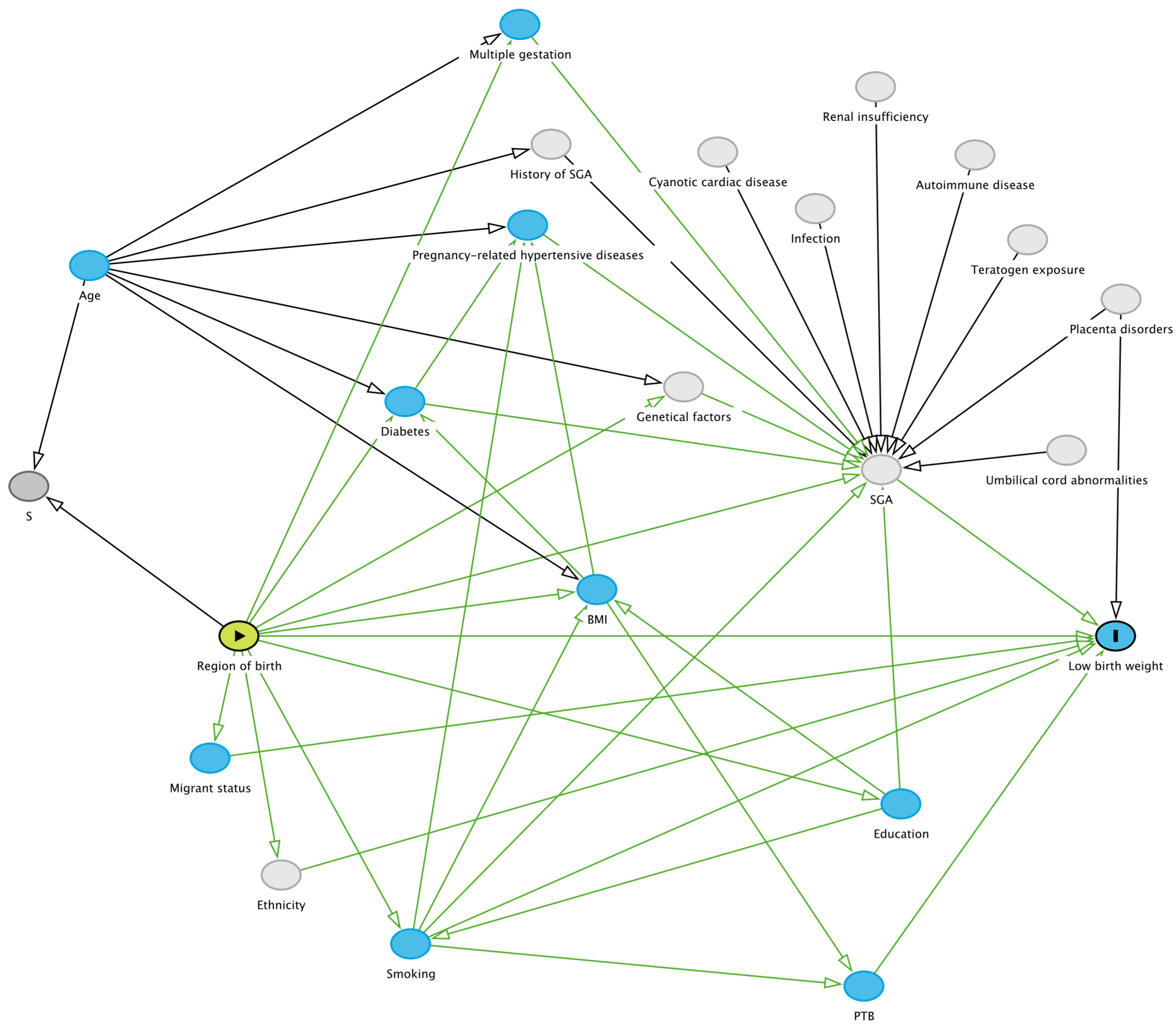

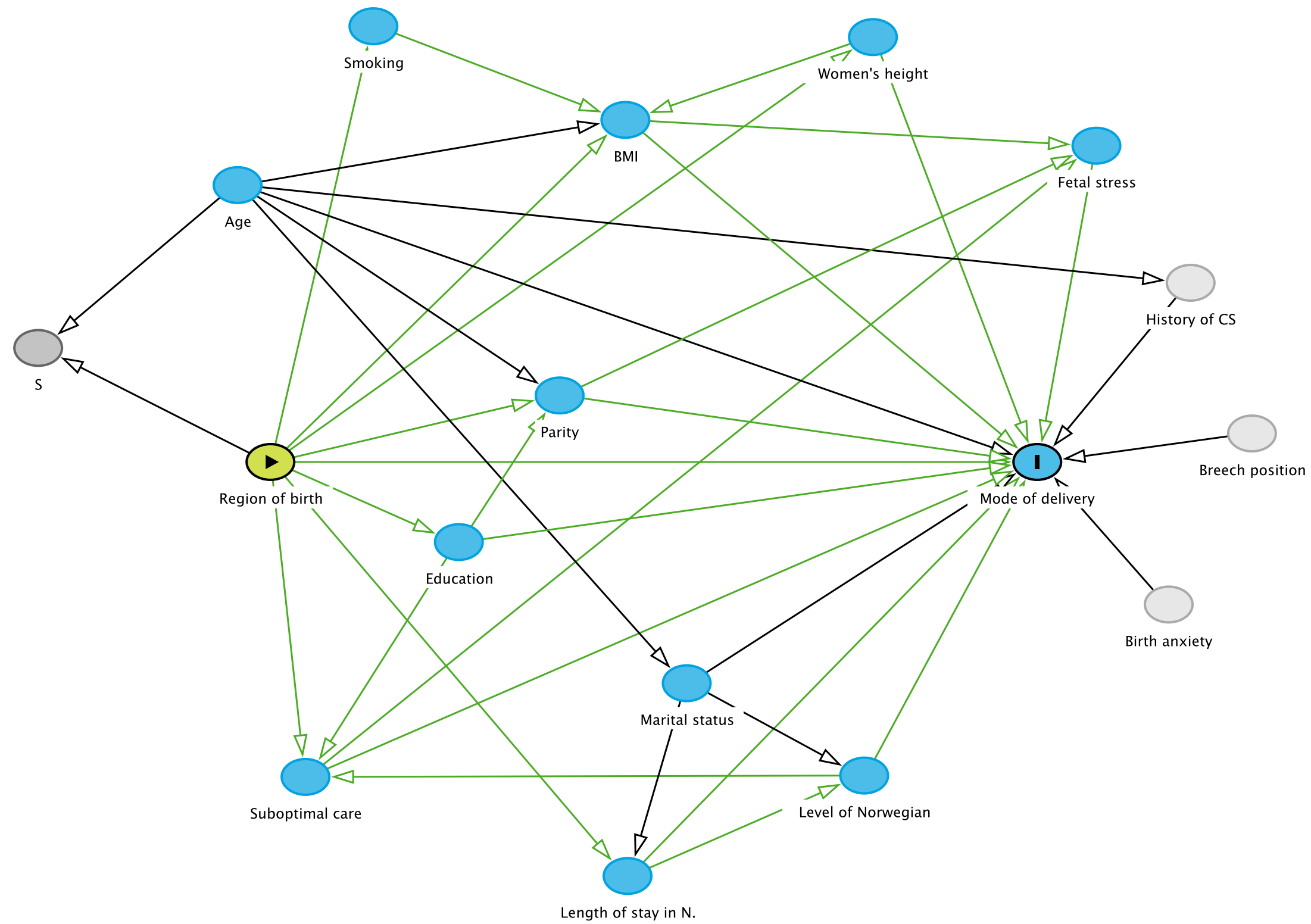

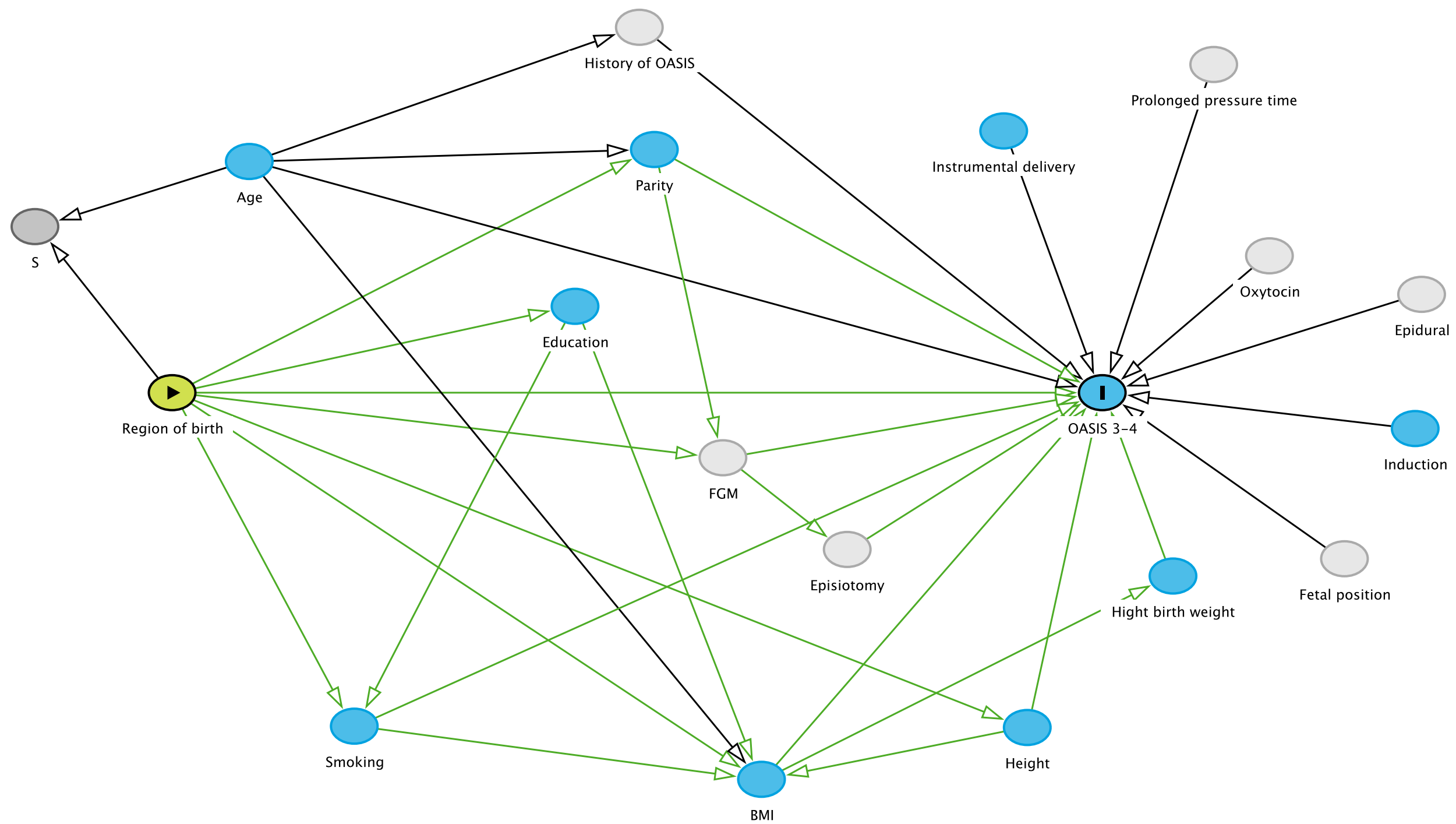

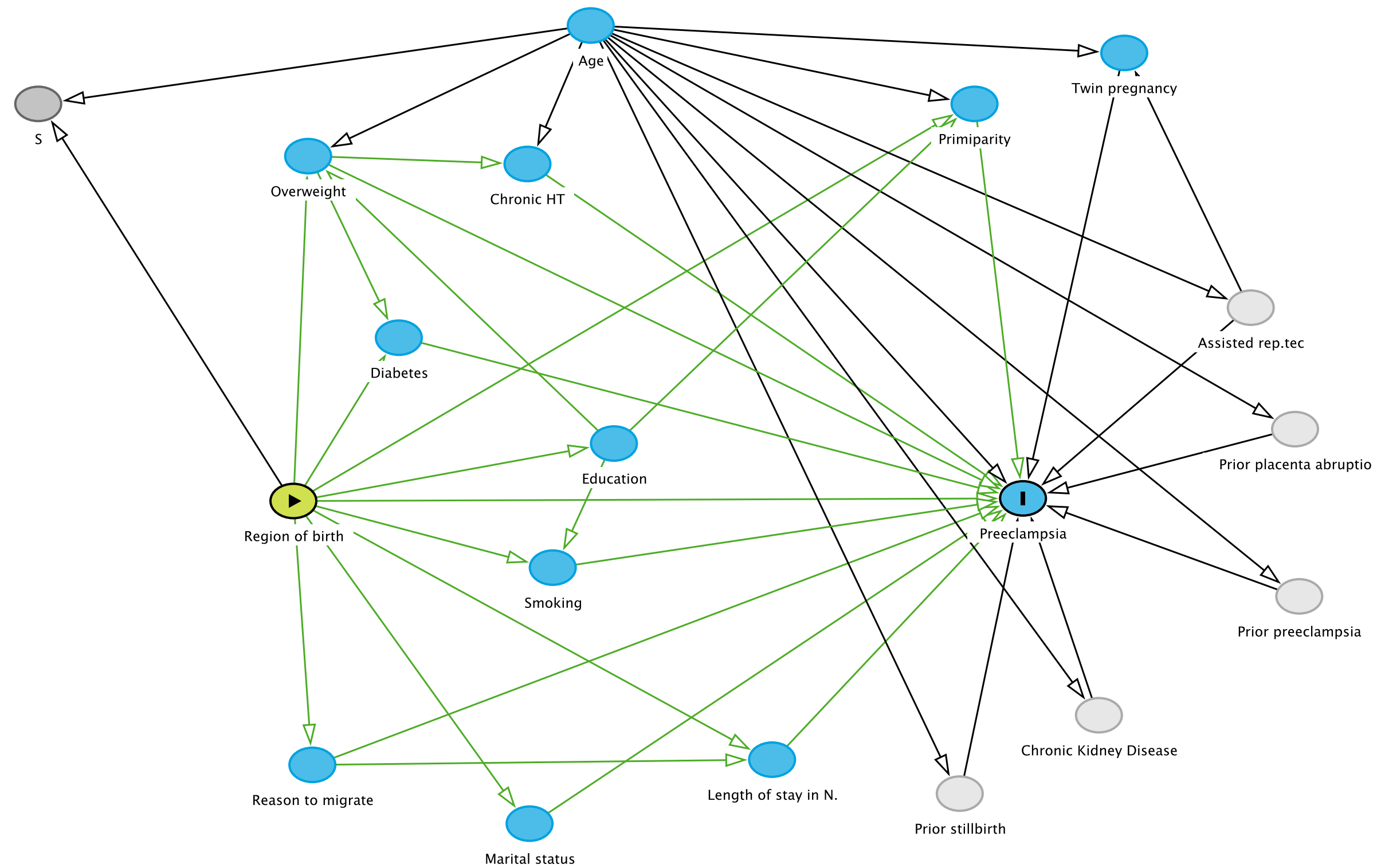

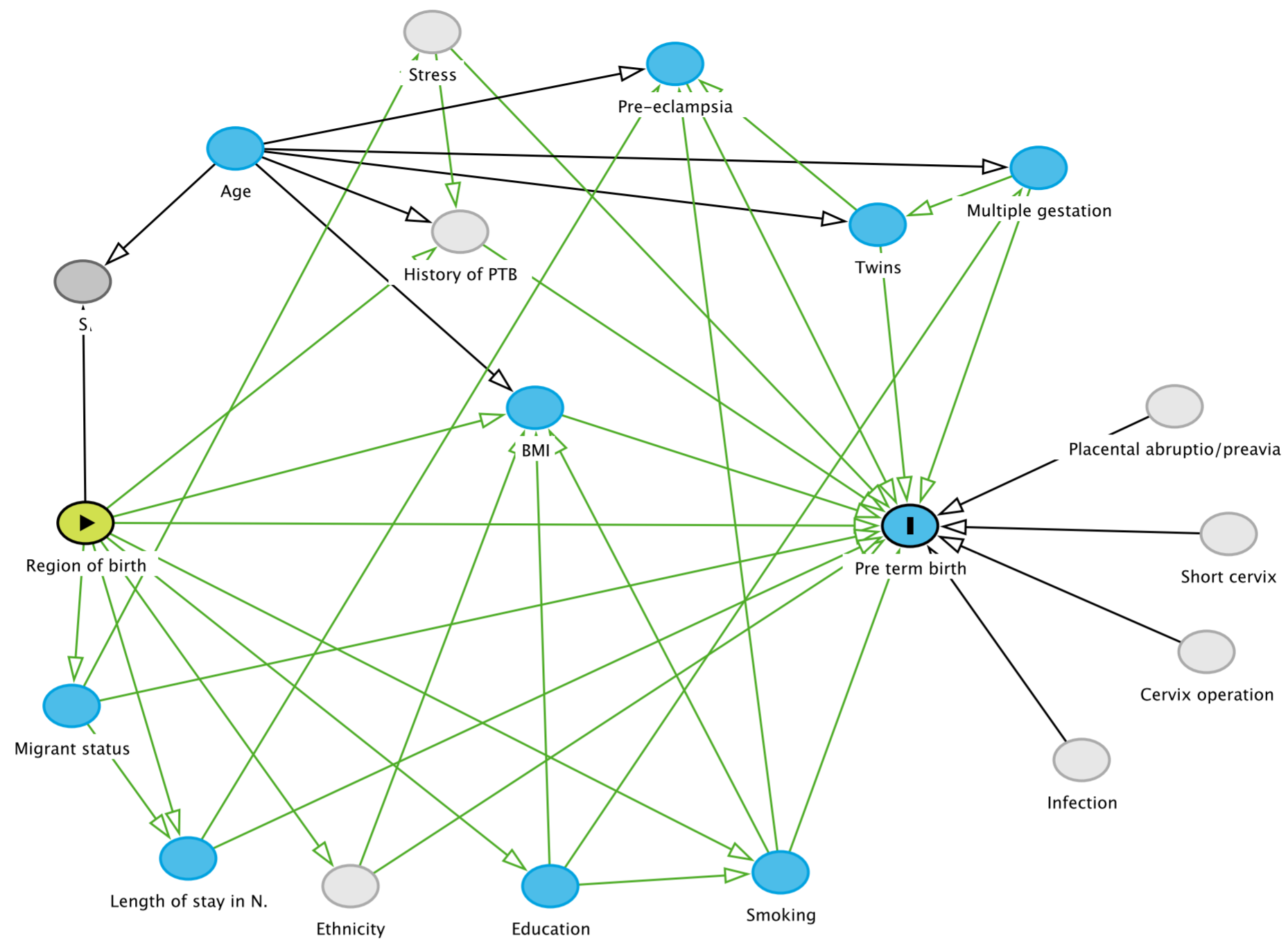

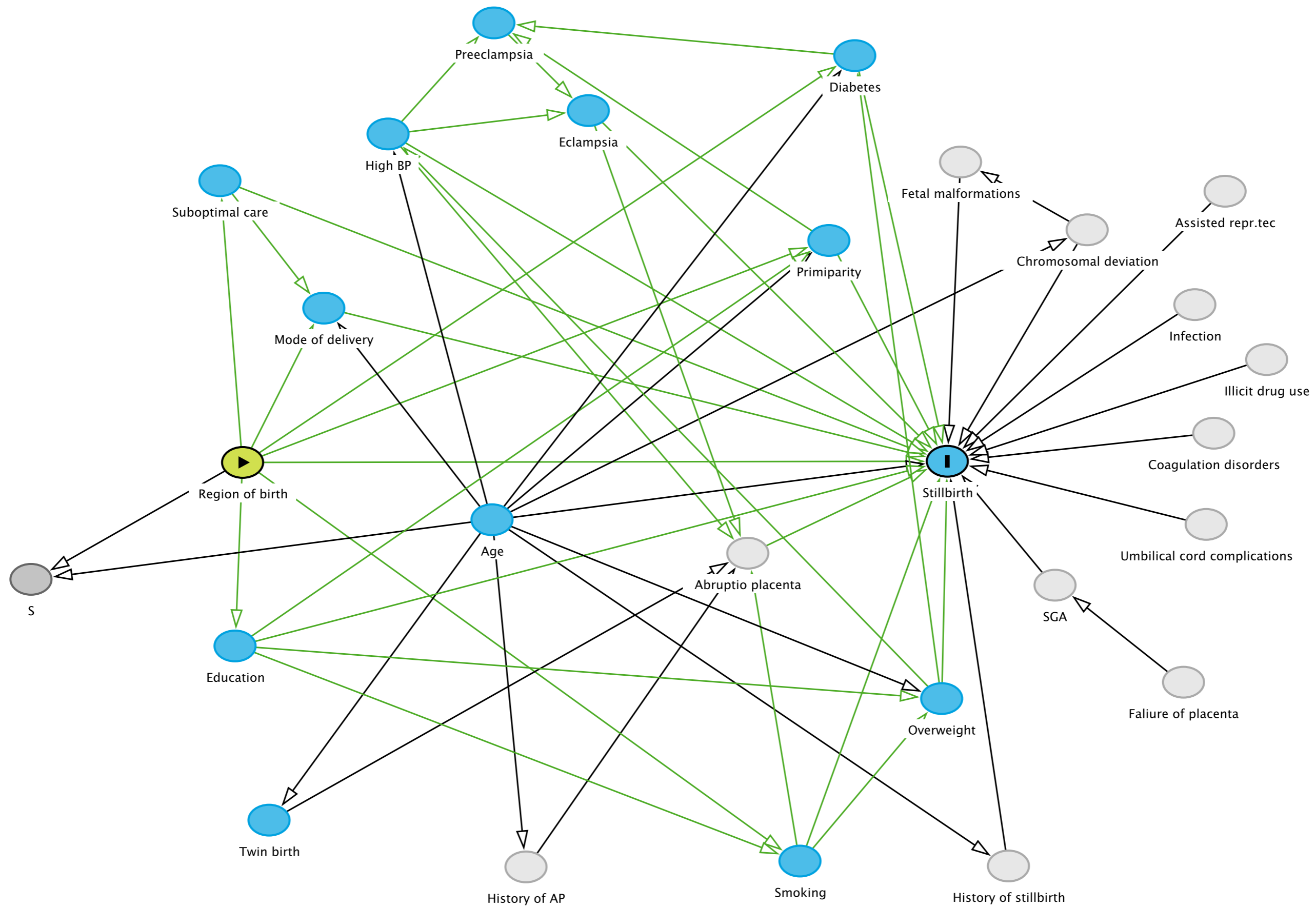

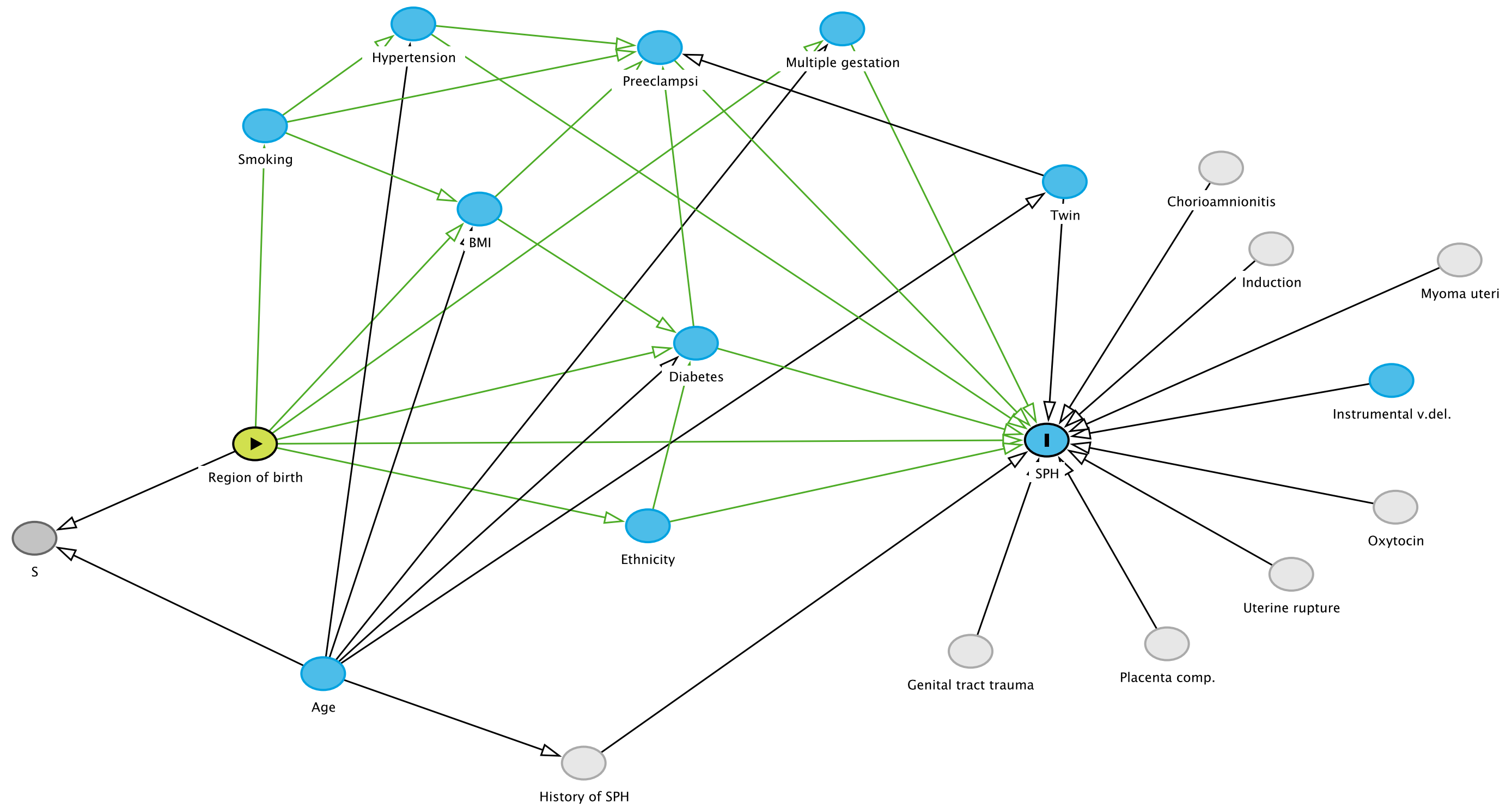

Supplement: Supplementary file 1 — Additional file 1. Directed Acyclic Graphs. [file 12884_2022_5112_MOESM1_ESM.pdf]
